# Transmissible colistin resistance encoded by *mcr-1* detected in clinical Enterobacteriaceae isolates in Singapore

**DOI:** 10.1038/emi.2016.85

**Published:** 2016-08-17

**Authors:** Jeanette WP Teo, Ka Lip Chew, Raymond TP Lin

**Affiliations:** 1 National University Hospital Department of Laboratory Medicine, Division of Microbiology, Republic of Singapore, Singapore 119074, Singapore; 2 National Public Health Laboratory, Ministry of Health, Republic of Singapore, Singapore 138623, Singapore

**Dear Editor**,

Following the recent description of transmissible plasmid-mediated colistin resistance encoded by *mcr-1* in clinical and veterinary *Escherichia coli* and *Klebsiella pneumoniae* isolates in China,^1^ several groups have reported *mcr-1* in colistin-resistant isolates from humans and food animals across Asia^2, 3, 4^ to Europe.^5, 6, 7, 8^ It is evident that *mcr-1* has disseminated globally.

We performed a prospective study in January 2016 using 306 consecutive clinical Enterobacteriaceae isolates collected from blood, urine and miscellaneous samples (swabs and pus). The species investigated were *E. coli* (*n*=166), *K. pneumoniae* (*n*=87), *Klebsiella oxytoca* (*n*=4), *Enterobacter* spp. (*n*=22), *Proteus* spp. (*n*=10), *Citrobacter* spp. (*n*=9), *Morganella morganii* (*n*=5), *Providencia rettgeri* (*n*=1), *Salmonella enteritidis* (*n*=1) and *Serratia marcescens* (*n*=1). Isolates were PCR-screened for *mcr-1*^1^ without prior knowledge of their antibiograms or colistin-resistance status. Three of these isolates (two *E. coli* and one *K. pneumoniae*) were *mcr-1* positive, with their full-length *mcr-1* gene matching the nucleotide identity of the first-described isolate exactly.^1^ Multilocus sequence typing (MLST) was performed (http://bigsdb.web.pasteur.fr/index.html). There was no evidence of nosocomial transmission, as the two *E. coli* isolates were of different sequence types (STs; Table 1).

The isolates were urinary specimens (Table 1). Our detection rate was estimated to be 0.98% (95% confidence interval of 0.3%–2.8%, Wilson score interval). This was very close to 1% *mcr-1* prevalence in China.^1^ Sensitivity testing was performed via E-test for polymyxin B and colistin and with the Vitek2 GNR257 card for all other antimicrobials. Phenotypically, the isolates were resistant to polymyxin B (minimum inhibitory concentration (MIC) 4 mg/L–24 mg/L) and colistin (MIC 4 mg/L–8 mg/L) but were sensitive to carbapenems. There was a variable sensitivity to third-generation cephalosporins and piperacillin-tazobactam (Table 1). PCR screening for carbapenemases (*K. pneumoniae* carbapenemase (KPC), metallo-β-lactamases (New Delhi Metallo-β-lactamase (NDM), verona integron-encoded metallo-β-lactamase, imipenemase), class D carbapenemases (oxacillinase (OXA)-23, OXA-48-like)) and broad- and extended-spectrum β-lactamases (BSBL and ESBLs) was performed.^9^ Only narrow-spectrum β-lactamase (TEM)-1 BSBL was detected in the *E. coli* isolates (Table 1). This was noteworthy because *mcr-1*-positive isolates have been found to be associated with cefotaximase (CTX)-M-like ESBLs.^4, 5, 6^ Furthermore, in our isolates, carbapenemase genes were not carried with *mcr-1*, which contrasts growing reports in which *mcr-1* has been found together with *bla*_KPC-2_ and *bla*_NDM_ carbapenemase genes^7, 8^ and results in colistin-resistant isolates that are also carbapenem resistant.^7, 8^ Using liquid-mating assays, *mcr-1* was transferable to an *E. coli* recipient, J53, in all the clinical donor isolates. Transconjugants were selected on Luria Bertani agar containing 50 mg/L of sodium azide and 0.5 mg/L of colistin. All of the transconjugants were phenotypically resistant to colistin and polymyxin B. TEM-1 also transferred in two transconjugants (Table 1). PCR replicon typing^10^ indicated that the transconjugants of NM2 and NM12 could not be typed, while transconjugant NM4 carried IncF and IncI1. The genetic environment of *mcr-1* is variable and is not always associated with an upstream ISApl*1*.^7^ Here PCR mapping and sequencing based on the initial genetic environment^1^ showed that one isolate did not have a flanking ISApI*1* (Table 1). This suggests that the dissemination of *mcr-1* is likely facilitated by a diverse range of mobile genetic elements. We plan to commence full-genome sequencing in the near future to characterize the plasmids and mobile elements in detail. Because Singapore has limited farming and agricultural activity, *mcr-1* is less likely to be acquired through contact with local livestock; although not yet conclusively proven, it appears that imported meat products and vegetables are more likely sources of *mcr-1*.^11, 12, 13^

## Figures and Tables

**Table 1:** Characteristics of *mcr-1*-positive clinical Enterobacteriaceae isolates in Singapore

**Isolate**	**Specimen**	**Species**	**Date of isolation**	**MLST**	**β-lactamases**	***ISApl1* associated**	***mcr-1* transmissible via conjugation**^a^	**MIC (mg/L)**^b^												
								**PB**	**COL**	**IMP**	**MEM**	**ETP**	**FOX**	**CAZ**	**AMP**	**PTZ**	**CIP**	**LEV**	**GEM**	**AMK**
NM12	Urine	*E. coli*	11/01/2016	ST460^C^	TEM-1	Yes	Yes, ≈10^−6^	4	4	≤0.25	≤0.25	≤0.5	≤4	≤1	≥32	64	2	4	<1	<2
NM4	Urine	*E. coli*	14/01/2016	ST156	TEM-1	No	Yes, ≈10^−7^	4	4	≤0.25	≤0.25	≤0.5	≥64	16	≥32	≤4	1	2	>16	<2
NM2	Urine	*K. pneumoniae*	13/01/2016	ST1535	TEM-1, CTX-M-15	No	Yes, ≈10^−3^	24	8	≤0.25	≤0.25	≤0.5	≤4	16	≥32	≤4	1	2	<1	<2
Transconjugant NM12	—	*E. coli*	—	—	TEM-1	—	—	2	2	≤0.25	≤0.25	≤0.5	≤4	≤1	≥32	128	2	4	≤1	≤2
Transconjugant NM4	—	*E. coli*	—	—	TEM-1	—	—	4	6	≤0.25	≤0.25	≤0.5	≤64	16	≥32	≤4	1	2	≥16	≤2
Transconjugant NM2	—	*E. coli*	—	—	Not detected	—	—	8	6	≤0.25	≤0.25	≤0.5	≤4	≤1	8	≤4	≤0.25	≤0.12	≤1	≤2
J53	—	*E. coli*	—	—	—	—	—	0.5	0.25	≤0.25	≤0.25	≤0.5	≤4	≤1	8	≤4	≤0.25	≤0.12	≤1	≤2

Abbreviations: amikacin, AMK; ampicillin, AMP; ceftazidime, CAZ; ciprofloxacin, CIP; colistin, COL; cefotaximase, CTX; ertapenem, ETP; cefoxitin, FOX; gentamicin, GEM; imipenem, IMP; levofloxacin, LEV; minimum inhibitory concentration, MIC; meropenem, MEM; polymyxin B, PB; piperacillin-tazobactam, PTZ.

a The conjugation efficiency is calculated as the number of transconjugants per donor cell.

b The interpretation of results of susceptibility testing were based on European Committee on Antimicrobial Susceptibility Testing (EUCAST) guidelines. For colistin, a susceptible breakpoint of ≤2 mg/L and a resistant breakpoint of>2 mg/L was applied. Because no interpretative criteria are available for polymyxin B, colistin breakpoints were applied.

c The Institut Pasteur MLST scheme was utilized. The following new allelic profile was obtained: *dinB* 22; *pabB* 21; *putB* 23; *trpB* 127; *icdA* 61; *polB* 109; *trpA* 41; *uidA* 164, and was assigned as ST460.
